# Comparison of Sensor Selection Mechanisms for an ERP-Based Brain-Computer Interface

**DOI:** 10.1371/journal.pone.0067543

**Published:** 2013-07-02

**Authors:** David Feess, Mario M. Krell, Jan H. Metzen

**Affiliations:** 1 Robotics Innovation Center, German Research Center for Artificial Intelligence, Bremen, Germany; 2 Robotics Research Group, University of Bremen, Bremen, Germany; University of Catania, Italy

## Abstract

A major barrier for a broad applicability of brain-computer interfaces (BCIs) based on electroencephalography (EEG) is the large number of EEG sensor electrodes typically used. The necessity for this results from the fact that the relevant information for the BCI is often spread over the scalp in complex patterns that differ depending on subjects and application scenarios. Recently, a number of methods have been proposed to determine an individual optimal sensor selection. These methods have, however, rarely been compared against each other or against any type of baseline. In this paper, we review several selection approaches and propose one additional selection criterion based on the evaluation of the performance of a BCI system using a reduced set of sensors. We evaluate the methods in the context of a passive BCI system that is designed to detect a P300 event-related potential and compare the performance of the methods against randomly generated sensor constellations. For a realistic estimation of the reduced system's performance we transfer sensor constellations found on one experimental session to a different session for evaluation. We identified notable (and unanticipated) differences among the methods and could demonstrate that the best method in our setup is able to reduce the required number of sensors considerably. Though our application focuses on EEG data, all presented algorithms and evaluation schemes can be transferred to any binary classification task on sensor arrays.

## Introduction

In recent years, the investigation and development of brain-computer interfaces (BCIs) based on the electroencephalogram (EEG) has gained a broad interest. This technology can be utilized in a wide range of applications. On the one hand, users may actively instruct devices like wheelchairs or spellers [1], [2]. On the other hand, passive systems can be used to surveil users and enhance the man-machine interaction in more subtle ways [3], [4]. In both cases, specific patterns in the EEG signals are exploited to predict the mental state of the user.

One major barrier for the usage of BCI systems in non-clinical applications is the complicated and time-consuming preparation. This procedure involves placing a large number of EEG sensor electrodes on the user's scalp and applying a conductive gel to each of them. The practicability of BCI systems could thus largely profit from a simplification of the preparation process. Recently, *dry electrode* systems that omit the conductive gel have been developed and promising results both in medical [5] and in BCI related applications [6] have been presented. This work follows a complementary approach that aims to enhance the usability by reducing the number of sensors (electrodes) that are required for achieving a satisfying performance of the BCI system. Therefore, we compare several sensor selection algorithms in the context of a passive BCI system that has the purpose of detecting a particular event-related potential (ERP) in single-trial [7].

Sensor selection aims at reducing the number of physical sensors that need to be placed. In contrast, a class of methods denoted as spatial filters tries to improve the signal quality by combining the readings from many physical sensors into a (typically smaller) number of ''virtual'' sensor channels. This is achieved by collecting the task-relevant information in a subset of the virtual sensors and discard the remaining ones which will contain mostly noise. This results in a reduction of dimensionality and thus potentially reduced computation time and a reduced risk of overfitting. However, it does not directly reduce the preparation time since all physical sensors are required for computing the virtual channels. Nevertheless, there are sensor selection algorithms which are closely related to spatial filters (see, e.g., [8]).

This paper gives an overview over the variety of existing sensor selection algorithms and presents them in a unified framework. The discussed algorithms range from selection schemes based on spatial filter coefficients over schemes based on the signal to signal-plus-noise ratio to a scheme, which performs sensor selection directly based on the classification performance of the respective sensor setting in the task of interest.

We present an empirical study in which we compare these sensor selection algorithms on EEG data using two different evaluation schemes. The first scheme denoted as *intra-session* is the one commonly used in the EEG literature: sensor selection and sensor evaluation are conducted on datasets from the same usage session (i.e., using the same sensor placement). In addition, we use an *inter-session* setup in which sensor selection and evaluation are performed on data from different usage sessions. In this setting the position of sensors, their electrical impedance, etc., may slightly differ, which in turn might influence the quality of the selected sensors. Furthermore, we compare the methods to meaningful baselines based on standard electrode caps and randomly selected sensor subsets of a given size.

Surprisingly, our empirical study shows that the sensor selection based on classification performance is not recommendable since it obtains worse performance than other criteria. Furthermore, our results clearly show that the intra-session evaluation scheme is not sufficient for assessing the quality of a sensor subset; it is, however, useful for comparing the relative quality of sensor selection methods.

In summary, the main contributions of this paper are that (a) several very different sensor selection algorithms are presented in a unified way and that (b) we empirically compare these algorithms and set them in relation to meaningful baselines.

## Methods

Typical approaches for the search of sparse sensor sets (constellations) consist of three components: firstly, one or more data sets using the full EEG sensor set have to be acquired. Secondly, most approaches require the definition of a validation function that specifies a real-valued score for each subset of sensors and thereby allows a ranking of sensor sets. Lastly, one needs a search strategy to find a set that performs well by means of this ranking function. Even though an exhaustive search approach to find the global optimum in principle is possible, it becomes already impractical for moderately large numbers of total sensors; e.g., there are more than 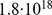 ways of picking 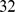 out of 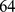 sensors. Thus, one needs to use search heuristics. A subset of the full sensor set for (hypothetical) subsequent measurement sessions can then be chosen using the search strategy such that the ranking score is maximized on the acquired data sets.

For evaluating the quality of the learned constellations, one would typically like to know how close the constellation's performance is to the optimal performance. This question is difficult to answer since the best performing sensor constellation is unknown and cannot be determined due to the gigantic number of constellations. Thus, we consider a different question which might give some alternative insights: How many randomly sampled constellations would one have to evaluate until one finds a constellation with equal or better performance than the one selected by the sensor selection method? For this we fit a distribution to the performance values of randomly sampled sensor constellations (see section ''Constellation Performance Distribution'').

### Overview: Sensor Set Ranking

Recently, a number of particular procedures and validation functions have been proposed. Interestingly, the validation functions operate on very different stages of the BCI signal processing chain–from the raw signal to filtered signals or even the classifier output. A brief overview is given in the following.

Barachant and Bonnet propose to rank sets of sensors based on the *Riemannian distance* between the class-specific covariance matrices [9]. This criterion is particularly efficient in the context of BCI systems that use the common spatial patterns (CSP) filter since CSP also relies on the covariance matrices as discriminating property of the data [10]. Lan et al. propose a sensor ranking based on the mutual information between the features derived from the sensor and the class labels [11]. In the context of BCIs that work with ERPs, the xDAWN algorithm can be utilized to compute particularly discriminative features [12]. xDAWN computes spatial filters that maximize the signal to signal-plus-noise ratio (SSNR) in a number of pseudo channels. Rivet et al. use the same concept also to rank sets of sensors according to the sum of their SSNRs [8]. This procedure will be discussed in more detail later in this manuscript (methods *SSNR
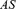
* and *SSNR
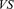
*).

The methods described so far can in principle be applied in any BCI scenario as they are not specific for any type of data processing. If, on the other hand, particular filters or classifiers are used, it may be possible to exploit them in order to compute a ranking from the estimated coefficients associated to a feature. Wang et al. apply this consideration to CSP weights [13] and we will come back to this type of procedures in the section on *xDAWN*, *CSP*, and *PCA* type methods. Lal et al. and Tam et al. go one step further in the processing chain and utilize the weights of a support vector machine classifier (SVM) in a similar fashion [14], [15]. We will later adopt this idea (methods *1SVM* and *2SVM*).

The key criterion for a comparison of the proposed methods in practice is the performance of the resulting reduced sensor BCI system–and as such the classification performance of the classification algorithm at the end of the data processing chain. An intuitive way of ranking sensor sets is therefore to evaluate the classification *performance* using the very set of sensors on a validation data set. Different choices for a ranking criterion based on the classification performance are imaginable. The straightforward approach we propose is to rank sensor sets according to the mean classification performance they obtain on several data sets. An alternative was proposed by Sanelli et al. [16]; the authors compute test errors for different sensor configurations and then evaluate through statistical tests whether each single channel contributes significantly to the classification performance or not. Then the p-values associated with these comparisons are used as scores. However, since p-values are random variables we argue that it is disputable to draw conclusions from the comparison of their values.

It shall be noted that some approaches deviate from the general procedure described so far. Farquhar et al. [17] and Arvaneh et al. [18], e.g., consider sparse variants of the CSP filter. In these works, the CSP model is extended by a regularization term that favors filters with contributions from few sensors. Sensors without or with small contribution to the spatial filters may be omitted for future sessions. A similar extension was proposed for the xDAWN spatial filter approach yielding the sparse xDAWN algorithm [19].

### Selected Ranking Approaches

In this subsection, we present a subset of the discussed methods in more detail and present them in a unified way which forms the basis for our empirical evaluation. The ranking function is always denoted by 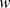 and a sensor set by 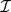.

#### Spatial Filter Ranking (*xDAWN*, *CSP*, *PCA*)

Linear spatial filtering is a common processing step in EEG data processing. Especially CSP and xDAWN have proven to be useful in different BCI scenarios [10], [12]. In addition to these two, we employ principle component analysis (PCA) [20] as a not primarily BCI-related, widely used spatial filtering method.

Essentially, in these filters the signals 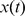 of different sensors are projected into a surrogate sensor space by multiplication with a filter matrix 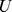: 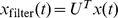. Each column of 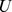 represents one spatial filter that generates one signal in the surrogate space as a linear combination of the original signals. What discriminates between different filter algorithms is the criterion for the determination of 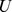. All algorithms considered here have in common that they come with an intrinsic ordering of the surrogate signals: it is expected that the *first* CSP or xDAWN channel carries the most information associated to one class and that the first PCA component carries the dimension of maximum variation. Based on these spatial filters, we obtain a straight forward ranking of the original sensors by summing up the absolute values of the filter coefficients associated with every individual sensor. More formally, a set 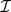 of sensors is ranked by
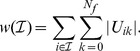
(1)


This ranking is motivated by the fact that the larger the absolute weight values of a specific channel for the first 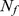 filters are, the higher is the channel's impact onto the most relevant 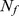 virtual channels. In our evaluation, we always consider the 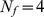 most relevant filtered channels. This value was chosen based on preliminary investigations.

The combination of this ranking method with the recursive backward elimination search strategy (see below) can be implemented particularly efficient since in each iteration, it is optimal to remove the sensor whose sum of absolute filter weights is minimal.

#### Signal to Signal-Plus-Noise Ratio (SSNR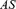, SSNR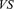)

Rivet et al. [8] propose to use the Signal to Signal-Plus-Noise Ratio (SSNR) as evaluation criterion in the context of ERP detection. They decompose the recorded signal 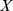 using least mean square estimation into three components: (i) the ERP component related to the stimulus of interest, (ii) responses common to every stimulus, and (iii) the residual noise. Based on this mixed effects model, the SSNR 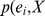) of the 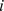-th sensor in 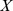 is defined as the ratio of the ERP component's energy to the recorded signal's energy. The first evaluation criterion they propose, the SSNR in the actual sensor space, rates a set of sensors 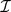 as the sum of the SSNR of the individual sensors in 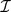:
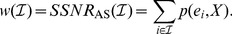
(2)


Note that this criterion cannot account for correlations in the individual sensors since each sensor's SSNR is determined in isolation. In order to account for correlations, a second criterion, the SSNR in the virtual sensor space, rates the same set of sensors as the sum of the SSNR of the first 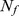 pseudo channels 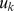. These 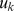 are generated using spatial filters that have been learned using the xDAWN algorithm on the data 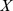 projected onto 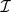 (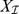). Formally,
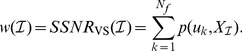
(3)


For further details, we refer to the original paper by Rivet et al. [8].

#### SVM Coefficient Ranking (*1SVM*, *2SVM*)

Linear SVM classification bases on the evaluation of the scalar classification function 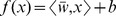 for each data sample 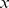 with some SVM parameters 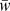 and 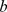. While 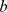 is a constant offset, the classification vector 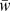 weights each individual feature similarly to a spatial filter as described above. If each feature originates from one single sensor, it is again possible to compute a sensor ranking by adding the absolute values of all weights of features belonging to each sensor. If this is not the case, e.g., if spatial filters were used, the feature weights would have to be distributed across the sensors that contribute to the feature.

In our analysis, we omit spatial filters when using this approach and consider two variants of SVMs: a standard SVM with 1-norm regularization (1SVM) or 2-norm regularization (2SVM). In comparison to their more commonly used 2-norm counterparts, SVMs with 1-norm regularization are known to operate on a reduced set of features [21]. They can thus be regarded as classifiers with intrinsic feature selection mechanism–a property that might be particularly advantageous in the context of sensor selection, provided that there is a direct relationship from a feature to a specific sensor.

Let 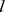 be the number of features per sensor and 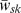 the weight of the linear SVM for the 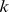-th feature belonging to sensor 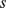. Now, a set 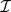 of sensors is ranked (analogously to spatial filter-based ranking) by
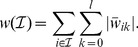
(4)


Because of the weight regularization, a SVM will only assign large absolute weight values 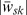 when the 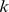-th feature of sensor 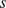 is is actually important for classification. Thus, ranking according to this criterion appears to be a sensible choice.

#### Performance-based Ranking (*Performance*)

The prevalent goal in BCI research is to provide a system that performs as well as possible in the context of a predefined application. From a machine learning point of view, the system's performance is equivalent to the performance of a classification algorithm that discriminates between specific commands or mental states. The most directive way to assess the effect of a change to the BCI system (like, e.g., changing the set of used sensors) is therefore to evaluate how it affects the classification performance in terms of a suitable performance metric. We use the balanced accuracy to measure the classification performance. The balanced accuracy 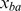 is the mean of true positive rate and true negative rate:
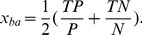
(5)


To be able to assess the performance, a validation data set is required so that the classifier can be trained and validated on different data. We use a 5-fold cross validation scheme to this aim. Further on, we propose to rank the sensor sets according to the mean performance they effect, i.e.:
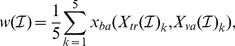
(6)where 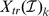 is the training set of the 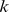-th fold of the cross validation containing only data from the sensors in 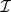, 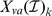 is the corresponding validation set, and 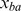 refers to the balanced accuracy the system achieves for this combination of training and validation data.

This method is computationally expensive, as a full cross-validation including repeated training of spatial filters and classifiers needs to be executed for every sensor set 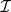. However, it may be expected that this approach yields the best results because the selection criterion (high classification performance) is exactly the same as the target criterion. Surprisingly, our results will show that this is not the case.

### Search Strategy: Recursive Backward Elimination

Given any of the sensor ranking methods described so far, one still has to define a strategy to find a set of sensors in the space of sensor constellations that corresponds to a high ranking. A popular approach to select 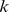 out of 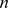 sensors is a *recursive backward elimination*. The algorithm was proposed as a feature selection strategy in a similar context as *recursive feature elimination* [22], and it can readily be transferred to sensor selection [8], [14], [15]. Starting with the full set of 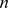 sensors, the idea is to evaluate the scores of all size 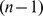 subsets. The set with the highest score is the starting point for the next iteration. We will stick to the recursive backward elimination procedure for the investigation in this paper. Alternatives to this heuristic include a forward search that adds one sensor at a time [11], a combination of forward and backward search [16], or evolutionary algorithms [23].

### Constellation Performance Distribution

We make the assumption that the performance, i.e., the balanced accuracy 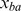, over the set 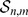 of sensor constellations with 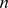 out of 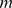 sensors follows a beta distribution (see Brodersen et al. [24] for a discussion):
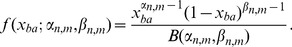
(7)


The parameters 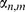 and 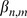 are determined by fitting the distribution to the balanced accuracy scores of randomly sampled constellations of size 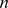. The probability of sampling a random constellation 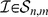 which obtains a balanced accuracy of at least 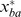 is obtained based on the cumulative density function of the beta distribution:
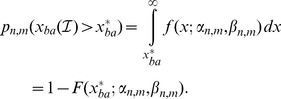
(8)


We will later evaluate this probability for the balanced accuracy scores obtained for constellations generated by the different sensor selection algorithms. This gives us an estimate of how many randomly sampled constellations would have to be evaluated until an equally good constellation is obtained. Furthermore, since 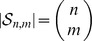, we can approximate the probability of randomly sampling the (unknown) best constellation as 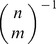.

### Implementation

All evaluations have been performed using a self-developed Python signal processing and classification environment (pySPACE) (pySPACE is scheduled to be released as open source in mid 2013. The configuration files used for this manuscript will than be available online). pySPACE is a modular software for the processing of large data streams that has been specifically designed for the empirical evaluation of EEG signal processing chains. The software enables the distributed execution of multiple processing tasks allowing us to run the analyses on a high performance cluster with 144 cores.

### Experimental Paradigm and Data Preprocessing

Our empirical evaluation is based on data acquired from a BCI system that belongs to the class of *passive BCIs*: the purpose is the gathering of information about the user's mental state rather than a voluntary control of a system [3]. Therefore, no deliberate participation of the subject is required.

This section is dedicated to the introduction of the BCI system and paradigm, followed by a description of the data processing we performed.

### Paradigm and Data Acquisition

The goal of the system is to identify whether the subject distinctively perceived certain rare *target* stimuli among a large number of unimportant *standard* stimuli. It is expected that the *targets* in such scenarios elicit an ERP called P300 whereas the *standards* do not [25]. Since passive BCIs aim at minimizing nuisance of their users, these systems will profit the most from a reduction of EEG sensors since the less sensors need to be applied, the less preparation time is required and the more mobile the system might become. So the users will probably be less aware of the fact that their EEG is recorded. Thus, basing the empirical evaluation on data from a passive BCI is a sensible choice.

**Figure 1:**
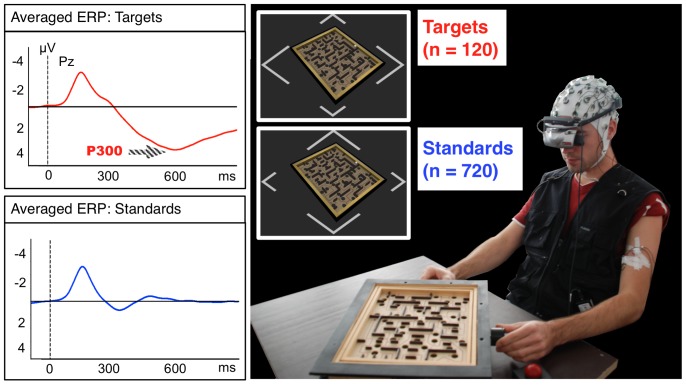
Labyrinth Oddball: The subject plays a physical simulation of a ball labyrinth game. He has to respond to rare *target* stimuli by pressing a buzzer and ignore the more frequent *standard* stimuli. The insets show the shape of the stimuli, which can be distinguished by the length of the edges. The graphs to the left depict the event-related potentials (ERPs) evoked by both stimulus types at electrode Pz. Both stimuli elicit an early negative potential attributed to visual processing, but only *targets* evoke an additional strong, positive potential around 600 after the stimulus.

The empirical evaluation was conducted on data recorded in the Labyrinth Oddball scenario (see Figure 1), a testbed for the use of passive BCIs in robotic telemanipulation. In this scenario, participants were instructed to play a simulated ball labyrinth game, which was presented through a head-mounted display. The insets in the photograph show the labyrinth board as seen by the subject. While playing, one of two types of visual stimuli was displayed every 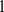 second with a jitter of 100. The corners arranged around the board represent these stimuli. As can be seen, the difference in the *standard* and *target* stimuli is rather subtle: in one case the top and bottom corners are slightly larger, in the other case the left and right corners are larger. The subjects were instructed to ignore the *standard* stimuli and to press a button as a reaction to the rare *target* stimuli.

Both *standard* and *target* stimuli elicit a visual potential as seen in the averaged time series in Figure 1 (strong negative peak at around 200 after the stimuli). Additionally, *target* stimuli induce a positive ERP, the P300, with maximum amplitude around 600 after stimulus at electrode Pz. It is assumed that the P300 is evoked by rare, relevant stimuli that are recognized, and cognitively evaluated by the subject.

The BCI only needs to passively monitor whether the operator of the labyrinth game correctly recognized and distinguished these stimuli. There is an objective affirmation of the successful stimulus recognition, because a button has to be pressed, whenever a *target* is recognized. However, since no feedback is given to the user, the testbed is well suited for evaluation of passive BCIs.

Five subjects participated in the experiment and carried out two sessions on different days each. A session consisted of five *runs* with 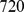 *standard* and 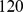 *target* stimuli per run. EEG data were recorded at 1 with an actiCAP EEG system (Brain Products GmbH, Munich, Germany) from 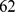 channels following the 10–10 layout (This system usually uses 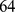 channels. Electrodes TP7 and TP8 were used for EMG measurements and are excluded here). For further details on the experimental procedure, we refer to Metzen et al. [26].

Aside from the study at hand, the data from the Labyrinth Oddball scenario have already been used for several investigations in a machine learning context, but with completely different focus. Topics include an improvement of the general processing flow [7], and the transfer of a processing flow onto an embedded hardware system [27]. Further, the behavior of spatial filters has been analyzed [28]–[30], and classifier threshold adaptation [31] and ensemble approaches were discussed [26].

### Ethics Statement

The study from which this data was taken has been approved with written consent by the ethics committee of the University of Bremen. Furthermore, subjects have given informed and written consent to participate. The study has been conducted in accordance with the Declaration of Helsinki. The subject of the photograph used for Figure 1 has given written informed consent, as outlined in the PLOS consent form, to publication of his photograph.

### Standard Signal Processing and Classification

This section briefly describes the signal processing and classification procedure used in the BCI system employed here. This procedure is used as the standard data processing within this BCI system and was chosen based on preliminary experiments; we will use it as reference and deviate from it only where the sensor selection methods require it.

All signal processing is performed on post-stimulus windows of 1 duration. The data from these windows are standardized (zero mean, unit variance) and low-pass filtered with a cut-off frequency of 4. Afterwards, an xDAWN filter is applied to the data. If more than 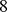 sensors were used, only the 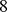 most relevant xDAWN channels are retained. Features are extracted from the filtered signal by fitting straight lines to short segments (These segments are cut out every 120 ms and have a duration of 400 ms) of each channel's data and using their slopes as features. The resulting features are again standardized (zero mean, unit variance) and then classified by an SVM classifier. During the training phase, the complexity parameter of the SVM is optimized using a grid search (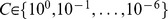) and 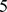-fold cross-validation. As the numbers of *standard* and *target* stimuli differ notably, different weights (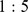) are assigned to both classes in the SVM. For the same reason, the *balanced accuracy* is used as measure for the classification performance: it is independent of the ratio of samples per class.

The individual sensor selection methods require the training and evaluation of different parts of the signal processing chain: the *SSNR* and *Spatial Filter* sensor selection algorithms can be applied for each run based on the signals after the low-pass filter and require no separate evaluation based on validation data. For the *SVM*-based methods, the entire signal processing chain has to be trained. In this case, the xDAWN filter is not used during the sensor selection in order to retain a straightforward mapping from SVM weights to sensor space. Again, no evaluation on validation data is required. The *Performance* method requires to train the entire signal processing chain, too; however, additionally, a validation of the trained system's performance is required. For this, the data from a run is split using an internal 5-fold cross validation (A leave-one-out scheme is not applicable in our scenario due to the high computational load). Each of the methods yields one sensor constellation per run for each session of a subject.

### Evaluation Scheme

**Figure 2:**
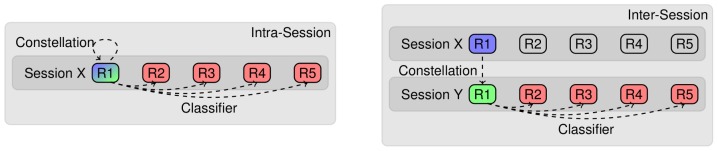
Intra-session and inter-session scheme. R1–R5 denote the runs from each experimental session. In the *intra-session* scheme (left), the sensor selection (blue) is performed in the same run in which the system is trained (green), and the evaluation (red) is performed on the remaining runs from that session. In the *inter-session* scheme (right), the sensor constellations are transferred to a different session of the same subject. Note that run and session numbering were permuted during the experiment so that in each condition, each run was used for sensor selection and training.

For evaluating the performance of a sensor selection method, three datasets are required: one on which the actual sensor selection is performed, one where the system (spatial filter, classifier, etc.) is trained based on the selected sensor constellation, and one where the system's performance is evaluated. From an application point of view, sensor selection should be performed on data from a prior usage session of the subject and not on data from the current one, on which the system is trained and evaluated (one would not demount sensors after a training run that are already in position). Since the selected sensor constellations are transferred from one usage session to another, this evaluation scheme is denoted as *inter-session* (see also Figure 2). The sensor constellations are thus evaluated on data from a different usage session with potentially different positioning of EEG sensors, different electrode impedances, etc. For the selected sensor constellation, the system is trained on data from one run of the session and evaluated on the remaining 4 runs. Thus, the inter-session scheme does not imply that classifiers are transferred between sessions but only that sensor constellations are transferred.

An alternative evaluation scheme, which is used frequently in related works, is the *intra-session* scheme (as depicted in Figure 2): in this scheme, the sensor selection is performed on data from the usage session itself; namely on the same run's data on which the system is trained later on. Thus, sensor constellations are not transferred to a different sessions and the influence of changes in EEG sensor positions and impedances is not captured. While this scheme is not sensible in the context of an actual application, it is nevertheless used often for evaluation of sensor selection methods because data of multiple usage sessions from the same subject may not be available. We perform the intra-session evaluation mainly to investigate to which extent its results generalize to the *inter-session* evaluation scheme.

To mimic an application case with a training period prior to an actual operation period, the evaluation is performed by applying a jackknife-like schema on basis of the runs from one session. In the *intra-session* scheme, one run is used for sensor selection and training of the classification flow, and the remaining four runs from that session are used as test cases. This is repeated so that each of the 5 runs is used for sensor selection/training once and results for our data set (consisting of 5 subjects with 2 sessions each) in a total of 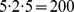 performance scores per selection method and sensor set size. In the *inter-session* scheme, we can perform the sensor selection on each of the five runs of the other session of the subject, and thus we obtain 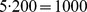 performance scores.

### Baselines

When reducing the number of sensors, it is often not clear what to use as a reference to evaluate the method's performance–there is no obvious *baseline procedure* and a certain loss in performance using a reduced number of sensors has to be expected. As we compare several different approaches, we can always compare them against one another. To draw an even more conclusive picture, we generated 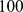 random electrode constellations for different numbers of sensors (The resulting performance values were also used to estimate the parameters 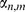 and 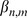 of the beta distribution in equation 8). This random selection does not incorporate any information from the data and should thus be outperformed by every approach that does so. As additional comparison, we evaluate two electrode constellations corresponding to commercialized EEG systems: one 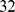 electrode 10–10 layout as used in the actiCAP EEG system and the original 10–20 layout with 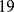 sensors.

## Results

**Figure 3:**
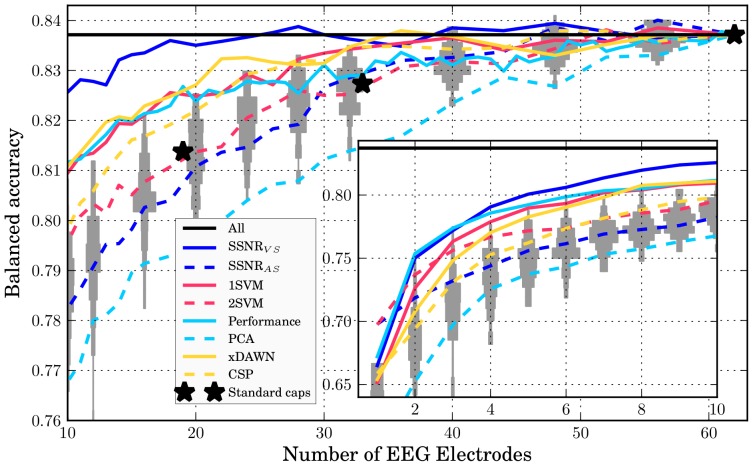
Intra-session evaluation of the classification performance versus the number of EEG electrodes for different sensor selection approaches. The horizontal line *All* is a reference showing the performance using all available 62 electrodes. The grey patches correspond to histograms of performances of 100 randomly sampled electrode constellations. The elongation in *y*-direction spans the range of the occurring performances and the width of the patches in *x*-direction corresponds to the quantity of results in that particular range. The three black stars represent widely accepted sensor placements for 19, 32, and 62 EEG electrodes. All other curves depict the mean classification performance over all subjects and cross validation splits. The results for 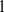-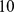 sensors are shown separately in the inset. By using an inset the curves in the main graphic appear less compressed.

Figure 3 shows the results for the intra-session scheme. At first it can be noticed that all standard caps perform essentially on chance level. The same is true for the *SSNR
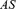
* and *2SVM* selection heuristics: for more than 5 sensors, both curves lie close to the center of the random selection patches. The *PCA* filter method performs even worse than random for a large range of constellation sizes. The *SSNR
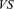
* method, the *xDAWN* filter, the *Performance* ranking, and the *1SVM* ranking deliver a performance considerably better than chance level for 30 or less sensors. The latter three perform nearly identically for the whole range on a level better than the expected chance level. The *CSP* method performs slightly worse than these methods for less than 20 sensors. For *SSNR
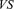
*, the mean performance remains on the baseline level of using all sensors down to around 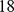 sensors and is remarkably better than any of the other heuristics.

**Figure 4:**
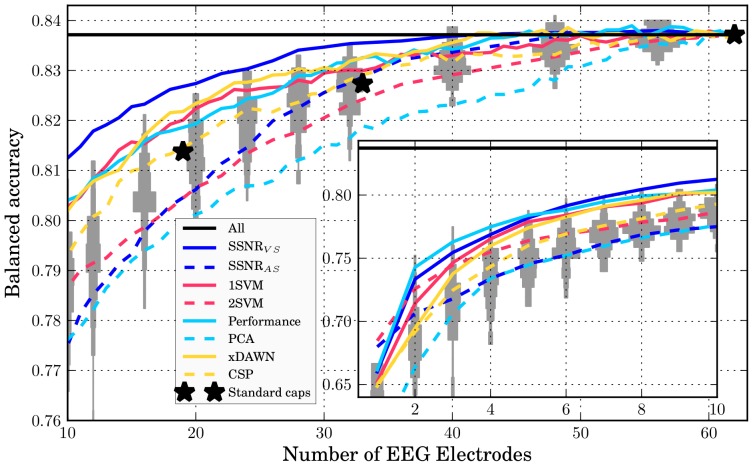
Inter-session evaluation of the classification performance versus the number of EEG electrodes for different sensor selection approaches. For more details, please see Figure 3.

In the inter-session results shown in Figure 4, all sensor selection methods drop in absolute performance compared to the intra-session scheme. Random constellations and standard caps are not effected by the type of transfer since they are not adapted to a specific session anyway. The relative order of the curves remains identical to the intra-session results. The performance of the best methods is still above or in the upper range of the random constellations, and *SSNR
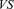
* still outperforms all random constellations in the relevant range.

**Figure 5:**
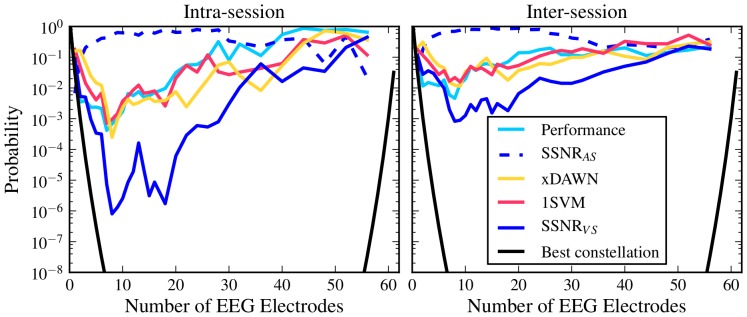
Probability of randomly sampling a sensor constellation that achieves an equal or better performance. Results are based on the assumption that balanced accuracy values for a fixed number of sensors are beta-distributed. The parameters of the beta distribution have been obtained by fitting values of 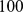 randomly sampled sensor constellations.

As described earlier in section ''Constellation Performance Distribution'' we also computed a measure of the likeliness of drawing a constellation at random, which performs at least as well as the constellation found by each of the sensor selection methods. Figure 5 shows the results for both the intra-session and inter-session scheme. It becomes evident that sensor selection has the largest utility if one wants to select between 7 and 20 out of the 62 electrodes. For the best method *SSNR
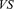
*, in the intra-session between 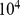 and 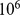 and in the inter-session approximately 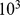 random constellations would have to be evaluated until one could expect to find an equally good constellation (Note that this does not imply that one would obtain equally good results when evaluating 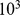 random constellations in the selection session and transfer the best one to the evaluation session; rather it means one would have to evaluate 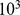 random constellations in the evaluation session). For more than 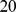 electrodes, the benefit of sensor selection gets smaller.

## Discussion

As the sensor selection of the *SSNR
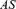
* and *2SVM* methods performs essentially equivalent to random selection, apparently these methods are not able to extract any useful information from the data. Possibly the *2SVM* would require an additional parameter optimization to be able to generalize well. A potential reason for the failure of the *PCA* could be that the sources with highest variance, which are preferred by PCA, might be dominated by EEG artifacts rather than task-related activities.

In accordance with the results of Rivet et al. [8], *SSNR
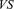
* performs considerably better than the relatively similar *SSNR
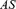
* ranker. This is most likely due to the fact that *SSNR
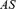
* cannot take redundancy between channels into account. *SSNR
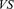
* accomplishes this by aggregating redundant information from different channels into a single surrogate channel via spatial filtering.

It is perhaps surprising that *SSNR
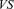
* performs better than *Performance*; we suspect that this might be caused by an overfitting of the sensor selection by *Performance* to the selection session. This effect might be reduced by using a performance estimate which is more robust than the mean, such as the median or the mean minus one standard deviation (to favor constellations with smaller variances in performance and less outliers). However, this issue requires further investigation.

For the inter-session scheme, the loss in performance of all methods in comparison to the intra-session scheme is expected. It results from the fact that due to day-to-day changes in brain patterns and differences in the exact sensor placement, different constellations may be optimal on different days–even for the same subject.

The fact, that the relative order of the results remains unchanged, however, indicates that a comparison of electrode selection approaches can in principle be performed without the effort of acquiring a second set of data for each subject. This facilitates the process of deciding for a particular sensor selection approach substantially. For obtaining a realistic estimate of the classification performance in future recordings with less sensors one needs a second, independent data recording session for each subject, however.

The results presented in Figure 5 show that there is clearly a big advantage sensor selection methods can offer. However, the specific choice of the method is crucial since some methods like *SSNR
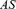
* perform essentially on chance level. For more than 20 electrodes, the benefit of sensor selection gets smaller; this is due to the fact that the variance in performance of random constellations gets smaller and thus, it gets more likely to sample a constellation that performs close to optimally.

### Conclusions

In this paper, we reviewed several sensor selection algorithms and compared their performance in the scenario of an ERP-based, passive, single-trial BCI [26]. We could demonstrate that the choice of the selection criterion is crucial for the maintenance of classification performance: Some algorithms generated sensor constellations which performed stably with around half the initial number of electrodes. Others, in turn, consistently performed worse then what could be expected from a random sensor selection. In our scenario, the most promising approach called *SSNR
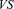
* is a sensor selection based on the maximization of the signal to signal-plus-noise ratio in combination with an xDAWN spatial filter [8]. *SSNR
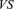
* allows to halve the number of sensors without loosing performance and performs considerably better than other selection methods or randomly sampled constellations. It is important to underline that *SSNR
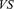
* is specifically tailored to ERP data–for other BCI systems, different approaches might prevail.

Beyond that we could show that one needs a second, independent data recording session for each subject to obtain a realistic estimate of the classification performance in future recordings with less sensors. On the other hand, we realized that this inconvenience is not necessary in order to decide in favor of a sensor selection method–the *inter-session* transfer does affect the absolute classification performance, but not the relative ranking of the selection approaches.

To improve performance in the *inter-session* transfer setting, it would be interesting to base sensor selection not only on one historic session but on several ones. This could allow to account for more of the inter-session variability during the sensor selection. Furthermore, it would be interesting to investigate whether good sensor constellations from different subjects show similarities, or whether it is even possible to generate suitable constellations that work for a wide range of subjects.
